# Survey data on livelihood assets, activities and outcomes of smallholder farm households in China's Loess Plateau

**DOI:** 10.1016/j.dib.2021.107638

**Published:** 2021-11-25

**Authors:** Qirui Li

**Affiliations:** a State Key Laboratory of Soil Erosion and Dryland Farming on the Loess Plateau, Institute of Water and Soil Conservation, Chinese Academy of Sciences and Ministry of Water Resources, Yangling 712100, China; b Leibniz-Centre for Agricultural Landscape Research (ZALF), EberswalderStraße 84, 15374 Müncheberg, Germany; c Africa Multiple Cluster of Excellence, University of Bayreuth, 95440 Bayreuth, Germany; d Climatology Research Group, University of Bayreuth, 95447 Bayreuth, Germany

**Keywords:** Land use, Payments for ecosystem services, Sustainable livelihood, Farm typology, Dryland agriculture, Social-ecological systems

## Abstract

Smallholders’ decisions on land use and their activities and strategies of livelihoods are the critical source of uncertainty in natural resource use and an essential determinant of sustainability challenges. This data article provides a selection of quantitative data from a questionnaire survey on livelihood assets, activities and outcomes of smallholder farm households in Yan'he Township, which lies in the middle part of China's Loess Plateau, one of the representative Grain for Green Project areas [1]. Data include land-use decisions and agronomic practices, fertilisation, use of pesticides, machine and irrigation, farm and non-farm activities, financial performance, and the levels of household income, wellness, and total consumption of food, energy, and education and health care. The survey also covered geographical, demographic and socioeconomic background information on the respondents and their perceptions, incentives, propensities and subjective wellbeing. The survey has supported a couple of research articles that build indicators and indexes for economic, environmental and socio-cultural sustainability dimensions and the resilience building of coupled social-ecological systems. The data presented in this article were analysed using descriptive and inferential statistics and provided at the Mendeley Repository. The data will assist studies on the interrelationships of smallholder livelihoods, ecosystem conservation, interventionist policy and market support, and community capacity building in sustainability science.

## Specifications Table

**Table:** 

Subject	Agricultural Sciences
Specific subject area	Agricultural Economics
Type of data	Primary data, Tables, Images
How data were acquired	Questionnaire survey
Data format	Raw, Analysed, Filtered (descriptive and inferential statistics)
Parameters for data collection	The survey data contain 247 households in China's Loess Plateau. The dataset consists of 242 valid observations after checking for missing values, potential errors, outliers and correlations. It covers the households in V-shaped valley areas and riparian (floodplain) areas with different altitudes and market distances.
Description of data collection	The data was collected through direct on-site interviews with household heads between February and May 2014. The author conducted the entire survey, who designed the semi-structured questionnaire for data accuracy and coherence.
Data source location	City/Town/Region: Yanhe Township in Ansai County of Yan'an City, Shaanxi Province Country: China Latitude and longitude: 36.48 and 109.22
Data accessibility	Dataset is uploaded on Mendeley Repository Name: Li, Qirui (2021), “Survey data on livelihood assets, activities and outcomes of smallholder farm households in China's Loess Plateau”, Mendeley Data, V1, doi:https://doi.org/10.17632/b2sjykvfgy.1
Related research article	Q. Li, H. Ma, Z. Xu, H. Feng, S. D. Bellingrath-Kimura, Balancing socioeconomic development with ecological conservation towards rural sustainability: A case study in semi-arid rural China, International Journal of Sustainable Development & World Ecology. DOI:https://doi.org/10.1080/13504509.2021.1990157

## Value of the Data

• This unique dataset about smallholders’ land-use practices and livelihoods in China's Loess Plateau represents the adaptation and transformation of local social-ecological systems (SES) under the rapid urbanisation and the implementation of a large-scale payment for ecosystem services (PES) program [1].
• The data will be helpful for researchers who would like to investigate local livelihoods, individual decision making, the effect of PES, feedback of human behaviour to ecosystem and policy interventions, and the transformation approaches towards sustainability in drylands.
• The data will be valuable to examine human behaviour and decision-making at the farm household level in response to policy interventions and changes in environmental and socioeconomic conditions, perform the impact assessment of land-use change, and conduct studies on resilience building and sustainability assessments.
• The data give insights into household demographics, local physical and geographic conditions, landholdings and transfers, household capital and assets, farming and non-farm activities and incomes, and household final consumption expenditure, characterised by sustainable livelihood principles and impact-feedback loops.
• In terms of policymaking and planning, key factors can be extracted to design farm management, community-based strategies and capacity building, and policy interventions and market support relevant to local agricultural production, food security, availability and consumption of ecosystem services, ecological conservation, rural infrastructure, social welfare and human capital, among others.

## 1 Data Description

**Fig. 1:**
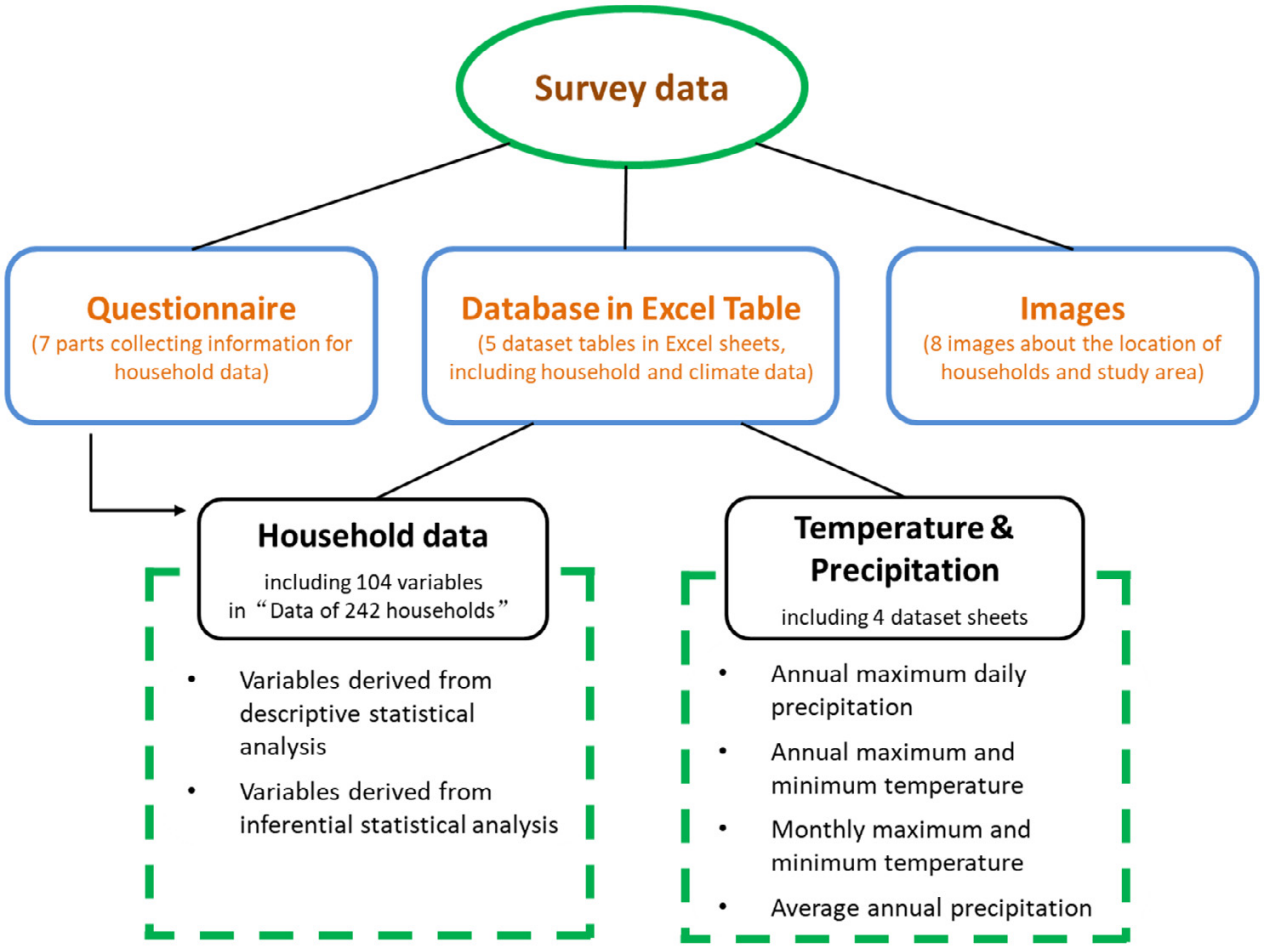
An overview of the survey data.

The data consist of five dataset tables, a semi-structured questionnaire, eight images about the location of the interviewed farm households and study area (Fig. 1). The household data table has 242 observations, including 104 variables that are generated from descriptive and inferential statistical analysis and connected to a question in the survey questionnaire. The questionnaire is divided into seven parts: 1) “Household Status” about the geographical and democratic information the household, 2) “Grain for Green Project” concerning the participation of the Grain for Green Project (GGP) implemented in 1999 to set aside sloping farmland (>25°) against soil and water erosion, 3) “Resource Availability” regarding the availability, allocation and utilisation of land, water, labour and capital, 4) “Cropping” including detailed information (e.g. price and timing) about seeds, sowing, planting, fertilisation, and the use of fertiliser, machine, pesticides, family and hired labour, and energy in terms of annual and perennial crops, 5) “Livestock Breeding” containing inputs and outputs of livestock breeding over various seasons and animal species. 6) “Off-farm Work” is the information of household members who engaged in off-farm work regarding age, gender, education, skills, wages, investments, duration, and destination, and 7) “Livelihood and Total Consumption” covering total consumption of food, energy, education and health care. The survey has already supported studies on sustainability assessment and the resilience building of SES [1], [2], [3], [4].

The data presented here has been used for the latest study on rural sustainability by balancing socioeconomic development with ecological conservation [5]. The data consist of variables with an uncountable set of values, binary response options (primarily coded as 1 = “yes” and 0 = “no”), and multivariate response options coded according to the number of alternatives and metric response options. Observations with missing values were dismissed. All variables are named in the dataset table and can be easily linked to respective questions in the survey questionnaire.

## 2 Experimental Design, Materials and Methods

### 2.1 Questionnaire development and survey sampling

The survey was conducted in 15 of 28 villages in Yanhe Township between February and May 2014. The Township represents the smallholder farm household in China's Loess Plateau regarding population density, topography, semi-arid climates, and the GGP scheme [1,2]. It followed a three-stage stratified sampling procedure, with a confidence interval of 6 at a 95% confidence level and a population (total households) of 3,390. Districts constitute the strata, covering two types of landscape—floodplain and V-shaped valley. Primary sampling units are villages. The survey villages account for 10,400 (62%) of the population of the Township, with different altitudes and market distances (Table 1). 20% of the households in each village were randomly interviewed.

**Table 1:** Description of survey sampling across villages, in Mean (Standard Deviation)

Village	Topography	Altitude (meters)	Distance to markets (km)	Farmland per capita (ha)	Ratio of farmland rented in	Ratio of farmland rented out	Variety of crop	Ratio of abandoned land to total arable land	Ratio of legume sown area to total sown area	Net income per capita (RMB)	Duration off-farm work (days)	Numer (share) of samples (%)
Houjiagou	V-shaped valley	1110.67 (10.97)	8.50 (0.01)	0.29 (0.21)	0.17 (0.34)	0	4.00 (1.00)	0.52 (1.56)	0.12 (0.15)	11033.33 (11421.47)	175.56 (223.43)	9 (3.72)
Siyaoxian	V-shaped valley	1197.46 (18.97)	12.15 (0.11)	0.24 (0.15)	0.07 (0.24)	0.05 (0.17)	4.08 (1.61)	0	0.18 (0.21)	22158.97 (21179.52)	186.92 (224.70)	13 (5.37)
Yanta	V-shaped valley	1114.86 (3.08)	8.30 (0)	0.27 (0.12)	0.16 (0.20)	0	4.43 (1.40)	0.05 (0.14)	0.15 (0.12)	7061.90 (6979.66)	174.29 (250.27)	7 (2.89)
Yujiahe	V-shaped valley	1190.93 (13.22)	16.55 (0.33)	0.39 (0.29)	0.05 (0.12)	0.07 (0.27)	3.43 (1.34)	0.02 (0.06)	0.01 (0.03)	19853.57 (18778.98)	14.88 (166.93)	14 (5.79)
Zhaiziwan	V-shaped valley	1173.57 (19.68)	13.24 (1.19)	0.34 (0.23)	0.07 (0.14)	0.01 (0.05)	3.57 (1.02)	0.02 (0.04)	0.02 (0.04)	16214.29 (22398.02)	155.00 (187.93)	14 (5.79)
Gaojiamao	V-shaped valley	1115.26 (21.23)	11.90 (2.84)	0.26 (0.26)	0.09 (0.20)	0.06 (0.21)	3.43 (2.06)	0.07 (0.21)	0.02 (0.05)	17302.17 (18759.90)	193.70 (215.23)	23 (9.50)
Yayao	V-shaped valley	1133.43 (12.73)	10.43 (1.52)	0.26 (0.12)	0	0.07 (0.27)	4.14 (1.79)	0.05 (0.15)	0.05 (0.06)	23484.52 (14550.87)	225.86 (214.49)	14 (5.79)
Zhuanyaogou	V-shaped valley	1049.73 (19.32)	3.04 (0.33)	0.17 (0.17)	0.23 (0.33)	0.06 (0.14)	2.00 (1.00)	0.24 (0.30)	0.02 (0.07)	14690.91 (14906.74)	124.55 (143.48)	11 (4.55)
Fangjiahe	V-shaped valley	1068.29 (14.63)	5.24 (0.02)	0.20 (0.13)	0.13 (0.19)	0.14 (0.30)	3.21 (1.58)	0.23 (0.28)	0.15 (0.18)	22642.86 (22324.90)	140.71 (175.91)	14 (5.79)
Zhifanggou	V-shaped valley	1053.00 (6.84)	9.74 (0.03)	0.24 (0.18)	0.55 (0.31)	0	3.50 (1.87)	0.08 (0.15)	0.16 (0.14)	48991.67 (75880.90)	221.00 (220.14)	6 (2.48)
Chafang	Riparian area	1052.15 (8.63)	7.23 (0.08)	0.06 (0.03)	0.07 (0.18)	0.22 (0.30)	2.05 (0.94)	0	0.43 (0.37)	23307.50 (14615.51)	299.50 (216.08)	20 (8.26)
Yunping	Riparian area	1038.53 (7.68)	8.43 (0.02)	0.06 (0.04)	0.16 (0.37)	0.30 (0.35)	2.05 (0.78)	0.44 (1.91)	0.27 (0.26)	17517.54 (14286.75)	268.03 (286.42)	19 (7.85)
Hougoumen	Riparian area	1030.80 (3.59)	7.20 (0.01)	0.09 (0.05)	0.10 (0.19)	0.26 (0.28)	1.15 (0.59)	0.31 (0.22)	0.20 (0.34)	22765.00 (33366.65)	281.94 (213.75)	20 (8.26)
Yanjiawan	Riparian area	1008.18 (9.22)	5.72 (0.02)	0.10 (0.09)	0.01 (0.04)	0.50 (0.40)	1.94 (1.34)	0.08 (0.23)	0.06 (0.14)	23464.71 (19749.78)	340.88 (270.00)	17 (7.04)
Lijiawan	Riparian area	1005.62 (6.01)	1.70 (0)	0.11 (0.10)	0.05 (0.12)	0.64 (0.38)	0.91 (1.24)	0.15 (0.33)	0.15 (0.07)	34224.74 (10612.06)	510.22 (191.89)	23 (9.50)
Yangjiagou	Riparian area	1052.15 (8.63)	7.23 (0.08)	0.06 (0.03)	0.07 (0.18)	0.22 (0.30)	2.05 (0.94)	0.23 (0.28)	0.43 (0.37)	23307.50 (14615.51)	299.50 (216.08)	20 (8.26)

A semi-structured questionnaire [6] was designed for information on household demographics, local physical and geographic conditions, landholdings and transfers, household capital and assets, farming and non-farm activities and incomes, and household final consumption expenditures. The interviews were carried out on-site with the help of a local guide (driver) in Mandarin Chinese. During the interview, Global Positioning System trackers were used to record the geospatial coordinates and elevations of household locations and main infrastructures (e.g., township centres and paved roads). All respondents were anonymised and plotted on soil maps to retrieve data on landscape elements. The soil maps were taken from the Soil Testing and Formulated Fertilization System (Ansai Agro-Tech Extension, Service Station 2010), including 4,314 soil samples tested in Ansai County in 2009.

The dataset includes 242 households after reviewing the data for missing values, potential errors, outliers and correlations. Nevertheless, the author acknowledges that the survey data cannot provide any information once the GGP is terminated in 2020. It would be updated after the COVID-19 pandemic.

### 2.2 Methods of data analysis

The database in Excel Table consists of five dataset tables in Excel sheets (Fig. 1), concerning the information of 242 households (i.e., “Data of 242 households”) and temperature and precipitation (in the other four sheets). The household data include 104 variables derived from the descriptive and inferential statistical analysis. Some variables are composed and calculated by using specific methods and parameters. For instance, total assets are items owned by the farm household for farming and non-farm work (Table 2). For valuing assets, cost value and market value of current, intermediate farm (1-10 years) and long-term (>10 years) assets are taken into account. The cost value is the initial and improvement costs of assets minus depreciation. Market value is an estimate of what the asset would sell for subtracting the associated selling cost. Current assets include cash and other assets that can be easily converted to cash, such as cash invested in growing crops and prepaid expenses. Intermediate farm assets are those assets with a useful life of 1 to 10 years (e.g., machinery and breeding livestock), whereas long-term farm assets have a useful life of more than ten years and usually can not be sold without disrupting the business (e.g., land and buildings).

**Table 2:** Item category in valuing total assets

Items	Farming	Non-farm work	Household
Current assets	GGP subsidy in 2013, received rent, allowance, crop for feed, crop for seed, crop for sale, animal for sale, self-seed, self-manure, mulch subsidy, fertiliser subsidy, the first purchase of animal, value of feed animal products, and value of pasture.	Wage	Saving, allowance of health insurance, credit, family gain, allowance, pension, and vegetables, fruits, eggs and animal meat for food.
Intermediate assets	GGP subsidy within ten years, pipe, water tank, pump, lamp, animal for breeding, machinery value, and animal stock value.	Value of equipment	Value of traffic tools.
Long-term assets	Grassland, forest, fallow and abandoned land, harvester, annual cropland, perennial cropland, seedlings, orchard, and tillage.	Investment	Value of drinking water system.

**Fig. 2:**
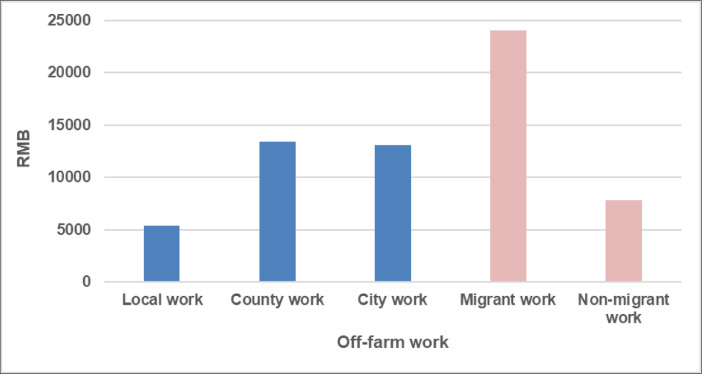
Net income of off-farm work per capita in 2013.

Moreover, crop frequency depicts the ratio of the months that farmland used in crop production to one year. It is the inverse of fallow farmland ploughed and harrowed but left for months without being sown. Farmers’ engagement in off-farm work (Fig. 2) is analysed by destination (i.e., work in local villages and communities, in the county and cities) and duration (i.e., migrant and non-migrant). In particular, migrant work means that a household member leaves for work for at least six consecutive months in a year. In addition, the calculation of total active workforce in person-day equivalents, farmland fragmentation, family labour in per-capita equivalents, livestock unit, net incomes and net margins, amount of nitrogen, phosphorus and potassium, liquidity and solvency, and protein intake is described in the previous publications [1], [2], [3], [4]. Farm prices, which vary across households, were taken into account for goods and services.

**Fig. 3:**
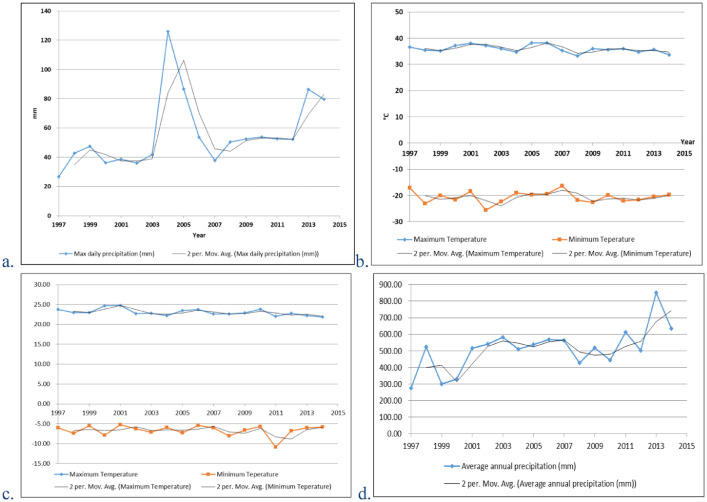
Temperature and precipitation data of the study area from 1997 to 2014. a. annual maximum daily precipitation and the two-period moving average; b. annual maximum and minimum temperature and the two-period moving average; c. monthly maximum and minimum temperature and the two-period moving average; d. average annual precipitation and the two-period moving average.

Data about temperature and precipitation (Fig. 3) of the study area is available from 1997 to 2014. It consists of annual maximum daily precipitation, annual maximum and minimum temperature, average monthly temperature, monthly maximum and minimum temperature, average monthly precipitation, and average annual precipitation. The data is observed and collected by the weather station of the Institute of Water and Soil Conservation, Chinese Academy of Sciences and Ministry of Water Resources.

## Ethics Statement

This paper does not involve studies with animals and humans. The work of this paper meets the ethical requirements for publication in Data in Brief (https://www.elsevier.com/authors/journal-authors/policies-and-ethics).

## CRediT authorship contribution statement

**Qirui Li:** Conceptualization, Methodology, Software, Data curation, Visualization, Investigation, Writing – review & editing.

## Declaration of Competing Interest

The field survey did not receive financial support from any institution. The author declares that he has no known competing financial interests or personal relationships that could have influenced the work reported in this paper.
